# Astaxanthin treatment ameliorates ER stress in polycystic ovary syndrome patients: a randomized clinical trial

**DOI:** 10.1038/s41598-023-28956-8

**Published:** 2023-02-28

**Authors:** Masoome Jabarpour, Ashraf Aleyasin, Maryam Shabani Nashtaei, Sara Lotfi, Fardin Amidi

**Affiliations:** 1 Department of Anatomy, School of Medicine, Tehran University of Medical Sciences, Pour Sina St, Tehran, 1416753955 Iran; 2 Department of Infertility, Shariati Hospital, Tehran University of Medical Sciences, Tehran, Iran; 3 Department of Infertility, Yas Hospital, Tehran University of Medical Sciences, Tehran, Iran

**Keywords:** Randomized controlled trials, Endocrine reproductive disorders

## Abstract

Astaxanthin (ASX), as a natural carotenoid compound, exists in various types of seafood and microorganisms. It has several possible beneficial therapeutic effects for patients with polycystic ovary syndrome (PCOS). Patients with PCOS also suffer from endoplasmic reticulum (ER) stress. In the present work, it was hypothesized that ER stress could be improved by ASX in PCOS patients. Granulosa cells (GCs) were obtained from 58 PCOS patients. The patients were classified into ASX treatment (receiving 12 mg/day for 60 days) and placebo groups. The expression levels of ER stress pathway genes and proteins were explored using Western blotting and quantitative polymerase chain reaction. To assess oxidative stress markers, follicular fluid (FF) was gained from all patients. The Student’s *t* test was used to perform statistical analysis. After the intervention, ASX led to a considerable reduction in the expression levels of 78-kDa glucose-regulated protein (GRP78), CCAAT/enhancer-binding protein homologous protein (CHOP), and X-box-binding protein 1 compared to the placebo group, though the reduction in the messenger RNA (mRNA) expression level of activating transcription factor 6 was not statistically significant. However, ASX significantly increased the ATF4 expression level. GRP78 and CHOP protein levels represented a considerable decrease in the treatment group after the intervention. In addition, a statistically significant increase was found in the FF level of total antioxidant capacity in the treatment group. Based on clinical outcomes, no significant differences were found between the groups in terms of the oocyte number, fertilization rate, and fertility rate, but the ASX group had higher rates of high-quality oocytes, high-quality embryo, and oocyte maturity compared to the placebo group. Our findings demonstrated that ER stress in the GCs of PCOS patients could be modulated by ASX by changing the expression of genes and proteins included in the unfolding protein response.

*Trial registration* This study was retrospectively registered on the Iranian Registry of Clinical Trials website (www.irct.ir; IRCT-ID: IRCT20201029049183N, 2020-11-27).

## Introduction

Polycystic Ovary Syndrome (PCOS) is one of the most prevalent endocrine and metabolic dysfunctionsin women of reproductive age. It is the most common cause of anovulatory infertility, affecting 6–10% of these women^1^. PCOS is a heterogeneous disease that includes both endocrine and metabolic disorders. It has various symptoms, such as insulin resistance, hyperandrogenism, gonadotropin disorder, and anovulation dysfunction. The pathogenesis of PCOS is unknown; however, it is believed that this disease is linked to oxidative stress (OS), mitochondrial dysfunction, chronic low-grade inflammation, and metabolic disorders that impair normal ovarian function^2–5^. The endoplasmic reticulum (ER) is a critical organelle in all eukaryote cells that regulates the quality of secreted proteins^6,7^. Calcium homeostasis, protein folding, cell differentiation, lipid metabolism, and protein translocation are all controlled by ER homeostasis^8,9^. Nonetheless, the ER function capacity may exceed its normal limits under certain conditions, such as nutrient deprivation, acid–base instability, hypoxia, and reactive oxygen species (ROS) accumulation. These factors cause fluctuations that interfere with ER stability. Additionally, the accumulation of misfolded or unfolded proteins in ER causes ER stress induction^10–14^. ER stress is a critical local factor that interacts with OS and inflammation. The unfolding protein response (UPR) is activated in cells due to ER stress. UPR is a highly conserved process during evolution^15^. GRP78, a 78-kDa glucose-regulated protein, is a central factor in initiating UPR^16^. It activates 3ER transmembrane molecules, including RNA-dependent protein kinase (PKR)-like ER kinase (PERK), inositol-requiring enzyme 1 (IRE1), and activating transcription factor 6 (ATF6), to initiate downstream UPR processes^16^. The apoptosis pathway is triggered by prolonged or severe ER stress^17^.

Cell death caused by ER stress is mediated by CCAAT/enhancer-binding protein homologous protein (CHOP)^18^. The primary function of IRE1 is to activate the UPR-related gene via X-box-binding protein 1 (XBP1)^19^. Furthermore, the principal roles of PERK are to reduce protein translation via eIF2 phosphorylation and to control transcription via ATF4 phosphorylation^20^. ATF6 activates the nucleus’ transcription factor, after moving from ER into the Golgi^21^. All three major UPR pathways have been found to regulate the pro-inflammatory transcriptional program via transcription factors, such as activator protein-1 and nuclear factor-κB(NF-κB)^22^. UPR and ER are essential‌ in granulosa cells' physiological and pathological events (GCs)^23^.

UPR activation and ER stress play critical roles in the pathogenesis and progression of human diseases, particularly genetic disorders, autoimmune diseases, metabolic dysfunction, cancer, and neurodegenerative diseases^24,25^. Recent studies indicate that ER stress occurs in ovarian cells, influencing follicle formation, oocyte maturation, and ovulation^26–31^. Also ER stress has been linked to ovarian disease in a few studies. Excess androgen in GCs can activate the ER stress pathway, leading to apoptosis via death receptor 5^32^. It is still difficult to develop an effective treatment for PCOS patients. In addition to different therapies for PCOS, lifestyle and diet changes have been suggested in this regard^33^. Diet and dietary factors play a major role in disease management. It has been shown that antioxidant interventions can improve PCOS complications, such as hormonal imbalances and metabolic disorders^34^. As a natural carotenoid compound, astaxanthin (ASX) is present in various kinds of seafood and microorganisms. Pluvialis-extracted ASX has been found to be safe and well-tolerated in daily dosages of 2–12 mg by the US Food and Drug Administration (FDA)^35,36^. Natural ASX has not been reported to be hazardous at any dose or for any duration of time in human investigations^37^. Various beneficial biological effects and activities are exhibited by ASX, including immunomodulatory and anti-inflammatory activity, protection against UV damage, cardioprotective effects, alleviation of metabolic syndrome, prevention of neuronal damage, anti-diabetic activity, inhibition of cell membrane peroxidation, and anti-aging and anti-cancer activities^38,39^. Additionally, previous studies have revealed the suppression of ER stress by ASX^40,41^. Thus, the present work aimed to determine the impacts of ASX supplementation on ER stress markers and OS in the GCs of infertile PCOS patients.

## Methods

### Trial design

Infertile PCOS patients aged 18–40 years were enrolled in this randomized clinical trial (RCT). They all met the Rotterdam criteria^42^ and were advised to undergo intracytoplasmic sperm injection (ICSI). PCOS was the only endocrinopathy in all of them and oligomenorrhea with oligo-ovulation due to the higher prevalence rate was the main reason for the inclusion of these patients.

Patients were excluded from the trial if they fulfilled any of the following criteria:

Severe endometriosis (stages 3 and 4 in terms of the revised AFS-rAFS classifying the endometriosis), FSH > 10 mg/mL, hyperprolactinemia, Cushing’s disease, ovarian tumors, thyroid disease, severe male factor infertility (particularly non-obstructive azoospermia), drug history affecting ovarian function in the 3 months prior to the study (steroids and oral contraceptive pills [OCPs]), female infertility factors other than cervical and tubal factors, any autoimmune disease, systemic disorders like metabolic syndrome, severe obesity and malnutrition (body mass index [BMI] over 35), hyperlipidemia, diabetes, and cardiovascular disease.

The participants were recruited from patients who were candidates for the ICSI protocol at Omid Clinic, Tehran, Iran, between November 2020 and September 2021. Although male factor indication for ICSI utilization appears to be constant, non-male factor indications remain controversial^43^. Some have advocated for the universal application of ICSI to all oocytes, regardless of the cause of infertility^44,45^. Moreover, several studieshave shown that conventional insemination of defective oocytes does not result in fertilization, whereas the use of ICSI increases fertilization and improve clinical outcome^43,46^. On the other hand, previous studies have shown that oocyte quality is low in patients with PCOS^47,48^. In our center, it has also been seen that PCOS patients with more dysmorphic oocytes have a lower fertilization rate after in vitro fertilization (IVF) compared to ICSI; accordingly, we employed ICSI, even though there was no male factor. The Ethics Committee of Tehran University of Medical Sciences approved the project (code:IR.TUMS.REC.1399.340) in accordance with relevant guidelines/regulations. The study was registered on the Iranian Registry of Clinical Trials (IRCT) website (IRCT-ID: IRCT20201029049183N1). Only a part of the results of the clinical trial registered with IRCT is presented in this paper. Before the intervention, each participant signed the informed consent form.

### Intervention

ASX 12 mg/day (2 × 6 mg capsules; Astareal, Tokyo, Japan) was orally given to the participants in the treatment (ASX) group for 60 days. In the placebo group, the patients received 2 capsules containing edible paraffin every day for 8 weeks with the same appearance as ASX. The capsules were simultaneously produced by a similar company. According to a former study, the ASX dose was selected as 12 mg orally per day^37^. In addition to following their normal daily routine, patients had to notify the researchers of any changes to their activity level or diet. To monitor participants’ dietary intake or activity levels, a 3-day food diary was collected during the study from all participants (1 weekend day and 2 weekdays). The Nutritionist IV program was used to estimate dietary intake. Further, patients completed a validated form of the 7-item International Physical Activity Questionnaire (IPAQ) at the start and end of the trial. To ensure compliance during the trial, all participants received daily reminders to take their supplements and were also asked to return the empty supplement bottles.

### Randomization

**Figure 1:**
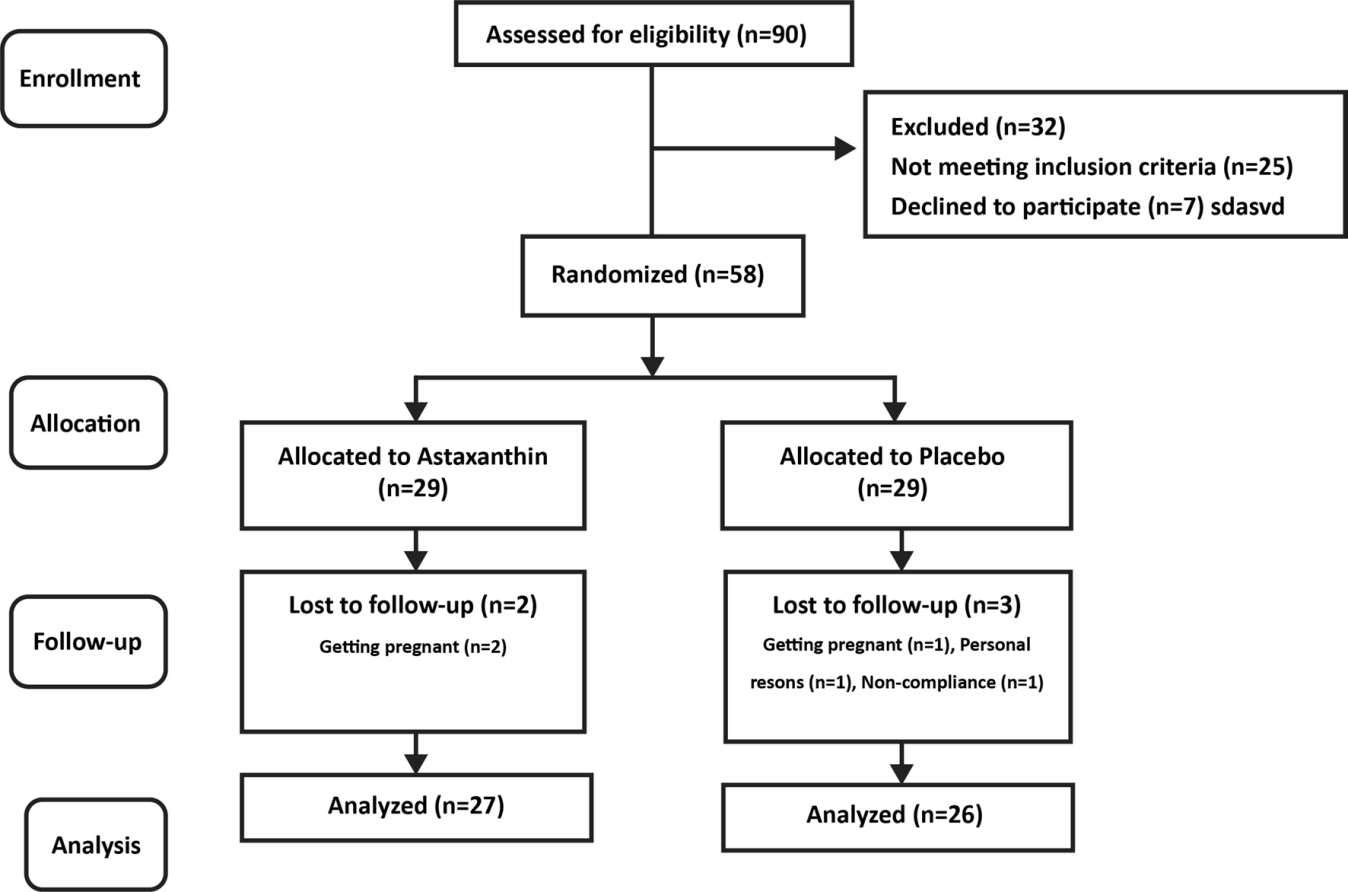
Summary of patient flow through the study.

First, 58 women were included in the work. The participants were randomly assigned to control (placebo) and intervention (ASX) groups through blocked randomization. The block size was 4 and contained letters A and B (representing the intervention and control groups). Sequentially numbered sealed opaque envelopes were used to conceal the contents. To avoid bias, participants were kept apart from other researchers during randomization. Figure 1 illustrates the study map. In this study, the statistician, patients, and investigator were unaware of the grouping, and the decipherer was not part of the research team. The ASX and placebo capsules were randomly distributed to the trial participants in identical bottles containing 60 capsules. It should be noted that the medicinal content of each bottle was labeled in the form of a code by someone other than the research team, and the research team was unaware of its interpretation.

### Ovarian stimulation protocol

An antagonistic regimen was administered to all patients (a flexible antagonist regime) for controlled ovarian stimulation. Prior to the ovarian stimulation cycle, all patients received OCPs (Ovocept LD, Abureihan, Iran) for 21 days. Briefly, 150–300 IU/day of recombinant follicle-stimulating hormone (Gonal-F, Merck Serono SA, Switzerland) was prescribed from the third day of the menstrual cycle. The optimum dosage was set considering the estradiol concentration and ovarian response. After monitoring the ovaries, when at least 2 follicles with the size of 14–15 mm were present, 0.25 mg/day cetrorelix acetate, Cetrotide (Merck Serono SA, Switzerland) was administered as the gonadotropin-releasing hormone antagonist. Cetrotide consumption was discontinued after reaching a diameter of 18 mm ≥ 2 for follicles; however, 10,000 IU human chorionic gonadotropin (hCG) was administered (Ovitrelle, Merck Serono SA, Switzerland), and oocytes were retrieved after 36 h through transvaginal ultrasound-guided aspiration^49^.

### Sample preparation

Samples were prepared according to a previous study^49^. Follicles were aspirated without blood contamination and flushing on the oocyte retrieval day. All FFs were collected from each patient and centrifuged at 3000* g* for 15 min. Furthermore, 5 mL of the supernatant was aliquoted and kept at − 80 °C for OS marker measurement. GCs were isolated by transferring the FF-derived pellet over 5 mL of Ficoll-Hypaque density gradient centrifugation (Lymphodex, Inno-Train, Germany). After centrifugation at 400* g* for 20 min, the cells from the interface were gathered and rinsed at 600* g* for 5 min. Moreover, 1 mL of phosphate-buffered saline (PBS; Sigma, Germany) with 1% bovine serum albumin (BSA; Sigma, Germany) was used for cell resuspension. Then, the cells were passed through a 40-μm cell strainer (BD Biosciences, CA, USA), and GCs were collected accordingly. Next, the cells were processed for the extraction of RNA and protein.

### Follicular fluid analysis

Follicular fluid (FF) was assessed for superoxide dismutase (SOD), total antioxidant capacity (TAC), and malondialdehyde (MDA) using colorimetric assay kits (Navand Salamat, Tehran, Iran). All OS parameters were detected in duplicate.

### RNA extraction and real-time polymerase chain reaction

Total RNA was manually extracted from GCs through a solution of RNX-PLUS (SinaClon, Tehran, Iran) based on the manufacturer’s protocol. The RNA concentration and purity were calculated via a spectrophotometer (Biochrom WPA Biowave, Cambridge, UK). Then, a complementary DNA (cDNA) synthesis kit (SinaClon, Tehran, Iran) was used to perform cDNA synthesis based on the manufacturer’s protocol. A RealQ Plus 2 × Master Mix Green (Amplicon, Denmark) was applied to define the gene expression levels. Additionally, polymerase chain reactions (PCRs) were conducted with specific primers for XBP1, ATF4, ATF6, GRP78, CHOP, and the glyceraldehyde‐3‐phosphate dehydrogenase as a housekeeping gene. All the reactions were performed twice by a Light Cycler 96 System (Roche, Germany). The messenger RNA (mRNA) expression of genes corresponding to the calibrator sample was calculated using the Livak method (2^−ΔΔCt^; Livak and Schmittgen, 2001). In the real-time PCR, 25 samples in each group were used for analysis. Table 1 presents the specific primer sequences.

**Table 1:** Specific primers used for real-time quantitative PCR.

Gene	Primer
GRP78	F: CTGTCCAGGCTGGTGTGCTCT R: CTTGGTAGGCACCACTGTGTTC
CHOP	F: GGTATGAGGACCTGCAAGAGGT R: CTTGTGACCTCTGCTGGTTCTG
ATF4	F: TTCTCCAGCGACAAGGCTAAGG R: CTCCAACATCCAATCTGTCCCG
ATF6	F: CAGACAGTACCAACGCTTATGCC R: GCAGAACTCCAGGTGCTTGAAG
XBP1	F: CTGCCAGAGATCGAAAGAAGGC R: CTCCTGGTTCTCAACTACAAGGC
GAPDH	F: CGC CAG CCG AGC CAC ATC R: CGC CCA ATA CGA CCA AAT CCG

*GRP78* Glucose regulated protein78*, CHOP* CCAAT/enhancer-binding protein homologous protein*, ATF4* activating transcription factor4*, ATF6* activating transcription factor 4*, XBP1* X-box binding protein 1*, GAPDH* glyceraldehyde-3-Phosphate dehydrogenase

### Western blot analysis

As described earlier^50^, RIPA buffer was employed to lyse cells to extract total protein. After centrifugation at 14,000 rpm and 4 °C for 20 min, a BCA Protein Quantification kit was used to measure the protein concentration based on the manufacturer’s instructions. An equal volume of 2X Laemmli sample buffer was added to the cell lysates. Next, the lysates (20 μg) were exposed to SDS-PAGE, followed by boiling for 5 min and transferring them to a 0.2-μm immune-Blot polyvinylidene difluoride membrane (Cat No: 162–017,777; Bio-Rad Laboratories, CA, USA). Then, the membranes were blocked with 5% BSA (Cat No: A-7888; Sigma Aldrich, MO, USA) for 1 h in 0.1% Tween 20. Subsequently, the membranes were incubated with Anti-GRP78 (Cat No: ab21685, Abcam), anti-chop (Cat No: ab194533, Abcam), and anti-beta actin-loading control antibodies (Cat No: ab8227; Abcam) at room temperature for 1 h. After washing the membranes with TBST, incubation was performed with a secondary antibody, goat anti-rabbit IgG H&L (HRP; Cat No: ab6721; Abcam). Next, incubation with enhanced chemiluminescence was conducted for the membranes for 1–2 min. The densitometry of protein bands was calculated using Gel Analyzer version 2010a (NIH, USA). Thirty-three samples in the Western blot, 17 in the treatment group, and 16 in the placebo group were used for analysis.

### Clinical data

Data on harvested oocytes, including the number of mature oocytes, the quality of the oocytes, and the percentage of high-quality oocytes, were obtained on the day of the puncture. It was determined that the high-quality oocyte rate was equal to the number of good-grade oocytes divided by the total number of retrieved oocytes × 100 for each participant. According to previous data^51,52^, 6 factors were used to assess the quality of individual oocytes, including oocyte morphology, range of possible oocyte sizes, ooplasm features, the structure of the perivitelline space (PVS), zona pellucida (ZP), and the morphology of the polar body. Oocyte morphology can be “worst” (characterized by an overall dark color and/or ovoid shape),“average” (characterized by a somewhat darker overall color and/or ovoid shape), or “best” (characterized by a normal color and shape). The range of possible oocyte sizes encompassed worst (abnormally tiny or huge, below 120 μ or larger than 160 μ), average (do not differ from best by more than 10 μ), and best (greater than 130 μ and less than 150 μ). The ooplasm feature was another intended factor (worst: extremely granular and/or vacuolated and/or demonstrating numerous inclusions; average: moderately granular and/or showing a small number of inclusions; best: showing no granularity or inclusions). The other factors were the structure of the PVS (worst: excessively large PVS, lack of PVS, or highly granular PVS; average: moderately enlarged/small/less granular PVS; best: best size PVS and the absence of granules in PVS) and ZP (worst: extremely thin or thick < 10 μ or > 20 μ; average:do not deviate from best by more than 2 μ; best: best zona > 2 μ and < 18 μ). The last factor was the morphology of the polar body, which can be the worst or multiple PBs (granular and/or either small or large PBs), average (fair but not excellent), and best (normal size and shape). The total oocyte score (TOS) was calculated by adding up the values assigned to each of the parameters; in addition, values of − 1, 0, and 1 represented the worst, average, and best, respectively. Oocytes may have a + 6 TOS and − 6 TOS at their highest and lowest levels, respectively. Oocytes were checked 16–18 h after ICSI to determine fertilization rates. Data on embryo development was gathered 2–3 days after ICSI, including number, quality, and high-quality embryo rate. In this study, the high-quality embryo rate was calculated as the number of high-quality embryos divided by the number of successful fertilizations × 100^53^ for each participant. The embryo quality is determined by its cell number (blastomeres), its fragmentation, and the presence of multiple nuclei, pits, and vacuoles^54^. In accordance with the ASEBIR (Association for the Study of Reproductive Biology) criteria, grade A and B cleavage embryos were classified as high quality based on these factors. The rates of chemical pregnancy (as determined by the hCG test) and clinical pregnancy (as determined by ultrasound to observe the gestational sac) were computed during the study.

### Statistical analysis

The data were presented as means ± SDs. The Kolmogorov–Smirnov test was used to confirm the normal distribution of data. Statistical analyses were performed by the paired Student’s *t*test or independent sample *t*test and Fisher’s exact test using SPSS version 22 (SPSS Inc., Chicago, Ill, USA). *P* values less than 0.05 were considered statistically significant. To compare gene expression as the primary outcome in this trial and due to a paucity of comparative research in this field, the standardized effect size was used to determine the sample size. Considering the large standardized Cohen’s effect size (d = 0.8), a type I error of 5% (a = 0.05), and a type II error of 20% (b = 0.2; power = 80%), the sample size was obtained 26 in each group. Considering the 10% loss to follow up, the sample size was calculated to be 29 subjects in each group.

### Ethics approval and consent to participate

The Ethics Committee of Tehran University of Medical Sciences approved the project (Ethics committee reference number: IR.TUMS.REC.1399.340) and all research was performed in accordance with relevant guidelines/regulations. Written consent was obtained from all participants.

## Results

**Figure 2:**
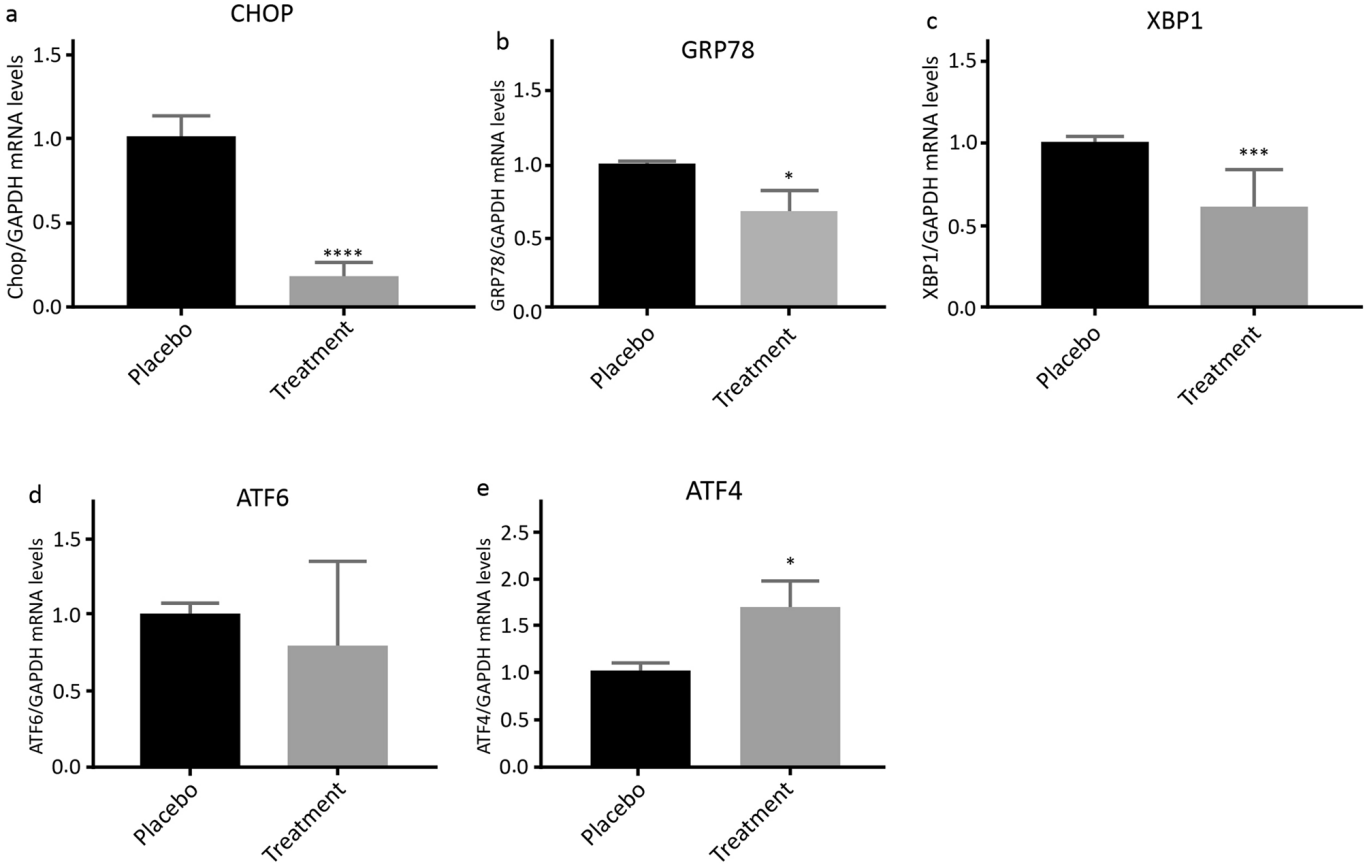
The fold changes levels of CHOP (**a**), GRP78 (**b**), XBP1 (**c**), ATF6 (**d**), and ATF4 (**e**) in GCs of placebo and treatment groups. Statistical significance (*p* < 0.05) was assessed by t-test. The results showed that fold changes levels of ATF4 was significantly increased in the intervention group (*P* < 0.05). After intervention, it was found that in the ASX group, the fold changes levels of CHOP, GRP78, and XBP1 were significantly decreased compared to the control group, while the reduction level of ATF6 was not significant between two groups (*P* > 0.05). *P*: placebo; T: treatment. Placebo: n = 25, Treatment: n = 25. Differences between groups; *p < 0.05, ***p < 0.001 and ****p < 0.0001.

**Figure 3:**
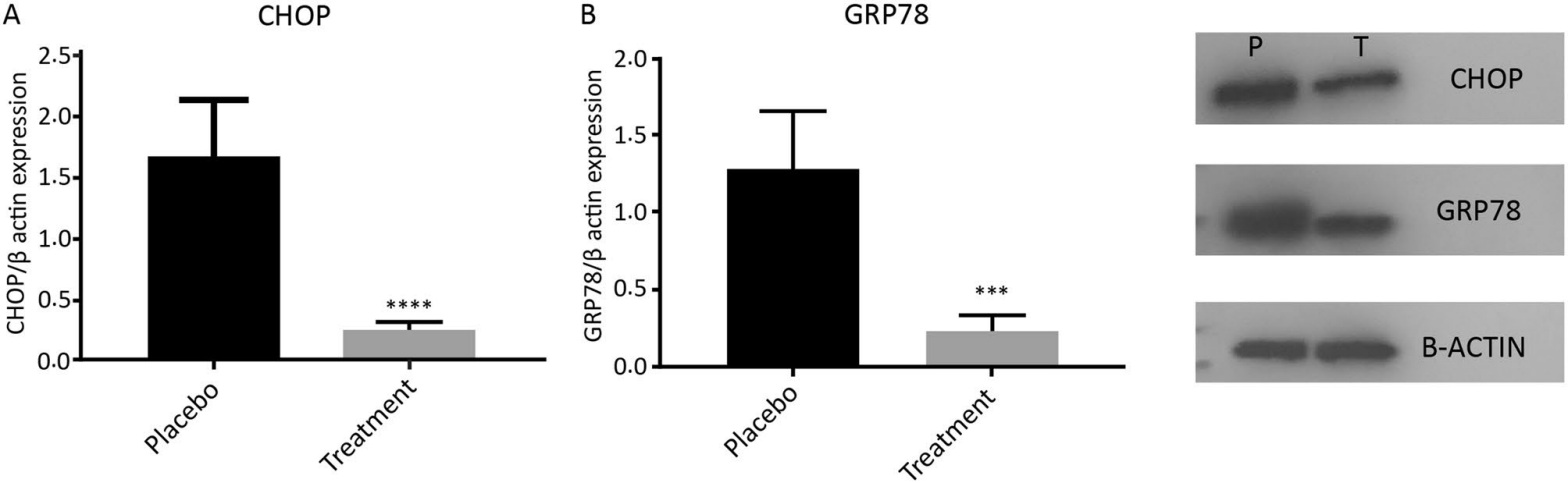
The protein expression levels of CHOP and GRP78 in the GCs of placebo and treatment groups. Western blot analyzed of the protein expression of Grp78 and Chop normalized to β-actin. Following intervention, the protein expression of Grp78 and Chop significantly reduced in treatment group compared to control group. Statistical significance (*p* < 0.05) was assessed by t-test. *P*: placebo; T: treatment. Placebo: n = 16, Treatment: n = 17. Differences between groups; ***p < 0.001 ****p < 0.0001.

**Figure 4:**
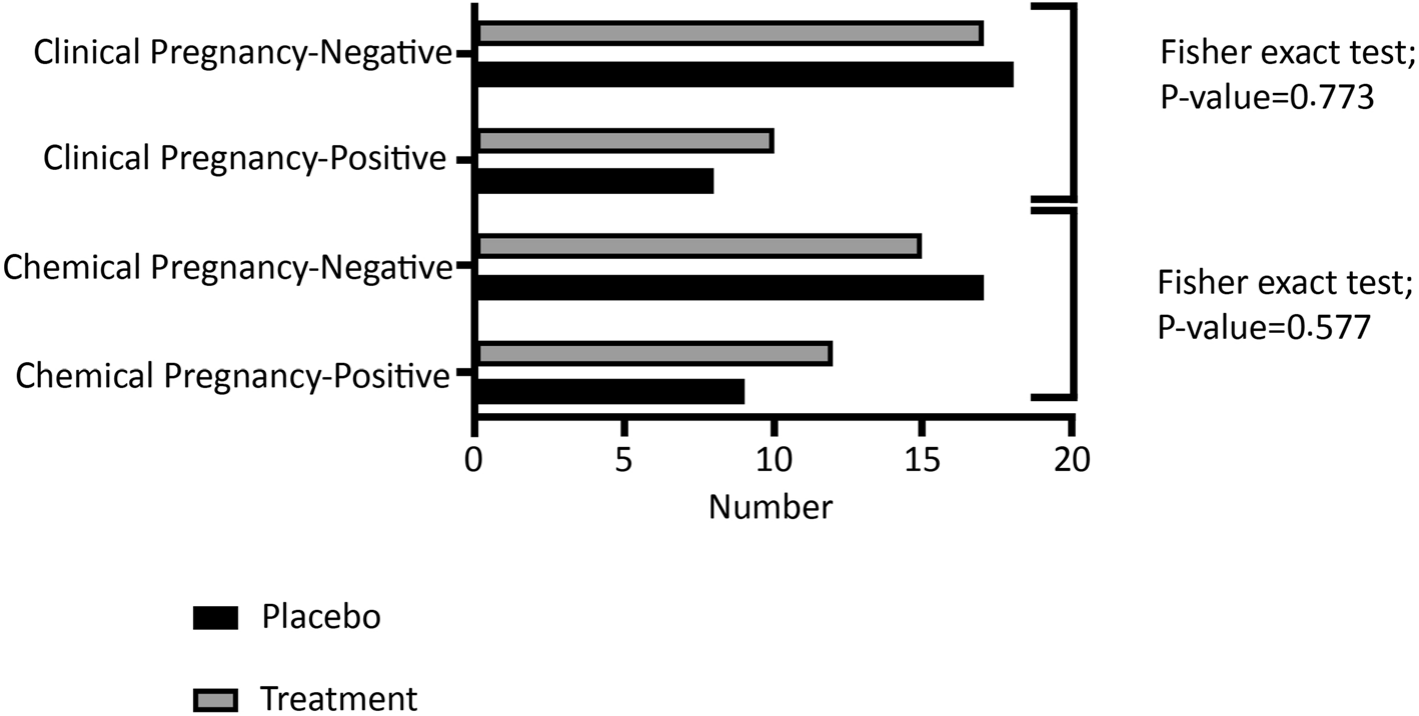
Comparison of clinical and chemical pregnancy rate between study groups. The chemical pregnancy rate was 44.44% (12/27) in the ASX group and 34.61% (9/26) in the placebo group (Fisher’s exact test; 1-sided *P* = 0.327, 2-sided *P* = 0.577). Moreover, the clinical pregnancy rate was 37.03% (10/27) in the ASX group and 30.76% (8/26) in the placebo group (Fisher’s exact test; 1-sided *P* = 0.424, 2-sided *P* = 0.773). Placebo: n = 26, Treatment: n = 27.

Generally, during the intervention stage, 5 subjects had to withdraw from the study, including 3 subjects in the placebo group (2 cases for personal reasons and 1 case due to pregnancy) and 2 cases in the ASX group (due to pregnancy). Finally, 53 patients participated in this study, including 27 and 26 cases in the intervention and placebo groups, respectively (Fig. 1). No adverse symptoms or effects with the ASX supplementation were reported by the participants during the trial, and they had good compliance with the intervention. No significant differences were also found in the duration of infertility, mean age, BMI, and hormonal profile between the treatment and control groups at the beginning of the study (Table 2). According to gene expression results, the expression levels of CHOP (*P* < 0.0001; Fig. 2a), GRP78 (*P* < 0.05; Fig. 2b), and XBP1 (*P* < 0.001; Fig. 2c) were significantly decreased in the treatment group than in the placebo group. Although in the treatment group, the mRNA expression level of ATF6 (*P* = 0.073; Fig. 2d) was reduced, this reduction was not significant statistically. However, the ATF4 expression level (*P* < 0.05; Fig. 2e) significantly increased compared to the placebo group. Moreover, the expression levels of CHOP and GRP78 protein significantly decreased after the intervention in the treatment group than in the control group (*P* < 0.0001; Fig. 3a and *P* < 0.001; Fig. 3b, respectively). Based on the results of the FF analysis (Table 3), a statistically significant increase was found in the TAC level in the treatment group (*P* < 0.05). Nonetheless, there was no considerable difference in the FF levels of MDA and SOD between the treatment and control groups (*P* > 0.05). According to Table 4, no statistically significant difference was detected in the number of retrieved oocytes, the number of embryos, and fertilization rates (*P* > 0.05), though the proportion of MII and high-quality oocytes rates were considerably higher in the study group than in the control group. Additionally, the embryo quality improved following ASX therapy. The results indicated that the rate of high-quality embryos was significantly higher in the study group than in the control group (*P* < 0.05). It was also found that the ASX group had a 44.44% chemical pregnancy success rate compared to the placebo group (34.61% success rate; (9/26, Fisher’s exact test; *P* = 0.577; Fig. 4). Additionally, the clinical pregnancy rate was 30.76% (8/26) in the placebo group and 37.03% (10/27) in the ASX group (Fisher’s exact test; *P* = 0.773; Fig. 4).

**Table 2:** Baseline parameters in individual group. Significance (*p* < 0.05) was assessed by t-test.

Variables	Mean ± SD Placebo (n = 26)	Mean ± SD Intervention (n = 27)	*P*-value
Age (years)	30.84 ± 4.84	30.36 ± 5.16	0.745
BMI (kg/m^2^)	26.24 ± 1.59	26.12 ± 1.56	0.802
Infertility duration (year)	3.386 ± 1.93	4.24 ± 2.02	0.147
Mean menstruation duration (day)	6.81 ± 0.9	6.48 ± 1.29	0.338
Mean menstrual cycle duration (day)	42.36 ± 10.05	44.12 ± 14.4	0.634
Baseline FSH (μIU/ml)	3.92 ± 1.11	4.19 ± 1.15	0.416
Baseline LH (μIU/ml)	9.01 ± 3.54	8.85 ± 3.13	0.874
Baseline Tes (ng/ml)	1.18 ± 0.56	1.24 ± 0.55	0.713
Baseline AMH (ng/ml)	9.21 ± 1.96	8.06 ± 3.01	0.132
Baseline PRL (ng/ml)	12.36 ± 1.8	13.14 ± 2.27	0.203

*BMI* body mass index, *FSH* follicle-stimulating hormone*, LH luteinizing hormone, Tes* testosterone*, AMH* anti-Müllerian hormone*, PRL* prolactin.

**Table 3:** Comparison of OS markers in FF of placebo and treatment groups.

Variables	Mean ± SD Placebo (n = 26)	Mean ± SD Intervention (n = 27)	*P*-value
SOD (U/ml)	173.4 ± 27.32	177.5 ± 19.85	0.55
TAC (mmol Fe2 + /l)	0.28 ± 0.06	0.32 ± 0.04	0.02*
MDA (μm/l)	2.55 ± 0.82	2.18 ± 0.89	0.14

Significance (*p* < 0.05) was assessed by t-test. Differences between groups.

*TAC* total antioxidant capacity, *SOD* superoxide dismutase, *MDA* malondialdehyde.

*p < 0.05.

**Table 4:** Comparison of clinical outcomes of placebo and treatment groups.

Variables	Mean ± SD Placebo (n = 26)	Mean ± SD Intervention (n = 27)	*P*-value
Number of retrieved oocytes	22 ± 4.88	21.26 ± 4.34	0.56
Rate of MII (mature) oocyte	68.81 ± 10.34	76.39 ± 8.07	0.004**
Rate of high quality oocyte	59.19 ± 10.45	67.9 ± 6.38	0.006**
Rate of fertilization	77.86 ± 10.13	81.24 ± 7.39	0.17
Number of embryos	11.04 ± 2.61	12.07 ± 2.49	0.14
Rate of high quality embryo	63.38 ± 13.68	70.24 ± 10.44	0.04*

Statistical significance (*p* < 0.05) was assessed by t-test. Differences between groups.

*p < 0.05, **p < 0.01.

## Discussion

Based on the findings of the present clinical trial on the effects of ASX on infertile patients with PCOS, the FF levels of MDA were reduced by ASX, while SOD and TAC levels represented an increase. Moreover, a 60-day course of ASX in the present work resulted in increased ATF4 mRNA expression levels. However, the expression levels of GRP78, CHOP, XBP1, and ATF6 mRNA were reduced compared to the placebo group. Our findings revealed that the protein levels of GRP78 and CHOP were also reduced after the pharmacological intervention. There is strong evidence that OS can result in ER stress^55^. ER stress was prevented by the administration of antioxidants, confirming the potential effects of OS on inducing ethanol-elicited ER stress^40,56,57^. In turn; OS is also induced by the accumulation of unfolded proteins in ER by various mechanisms. Mitochondrial oxidative phosphorylation is stimulated by the activated BiP, a key signal molecule in the ERstress pathway, to produce ROS as a by-product. Furthermore, the enzymes of the NADPH oxidase (NOX) family increase ROS production, particularly through Nox2 and Nox4 isoforms under ER stress^58–60^. The role of OS and ER stress in PCOS pathogenesis is highlighted by recent publications^23,61^. To determine whether ASX is effective in treating PCOS, the expression levels of mRNA and protein in the ER stress pathway were measured in our study. Our study focused on ER stress levels in the GCs of infertile PCOS patients who received ASX or not. Former investigations revealed an incremented expression of UPR genes such asATF4, ATF6, CHOP, and XBP-1 in GCs in PCOS patients^23^. According to our findings, the mRNA levels of GRP78, spliced XBP1, and CHOP were significantly decreased compared to the placebo group; however, the mRNA level of ATF4 was significantly increased in the ASX group. Various studies demonstrated that the activation of UPR and ER stress is associated with chronic diseases, including inflammation, diabetes, obesity, inflammatory bowel disease, neuromuscular inflammation disease, respiratory inflammation disease, PCOS, and arthritis^16^. Based on the results of studies on type 2 diabetes and insulin resistance, hyperglycemia has a close relationship with ER stress^61^. It was found that androgens can increase the GRP78 expression level^62^. Previous studies have confirmed the activation of ER stress by hyperandrogenism in PCOS^32^. ER stress includes some molecular cascades comprising some enzymes and transcription factors to restore homeostasis^16^. Based on a previous paper, GRP78 and UPR activator proteins (PERK, IRE1, and ATF6) increased in PCOS patients compared to healthy subjects^23^. Our results indicated that GRP78 mRNA and protein levels were significantly reduced by pharmacological intervention with ASX. A rise in the GRP78 expression in human diseases is an indicator of elevated protein misfolding conditions in ER, as well as a marker, to measure ER stress^16^. Treatment with ASX led to a reduction in the GRP78 expression level, indicating that ER stress conditions were reduced by interventions in PCOS patients, at least partially. The findings also showed a significant reduction in XBP1 mRNA expression after treatment with ASX in PCOS patients. Therefore, it is to be expected that ASX reduces XBP1 and thus decreases UPR target genes. It was concluded that the XBP1 expression level was reduced, while the ATF4 expression demonstrated a significant increase. According to former research, the expression of Nuclear factor E2-related factor 2(NRF2) is promoted by ATF4 by inducing genes included in antioxidant functions^63^. Our work revealed that ASX might have a role in NRF2 promotion by increasing ATF4. As a result of GRP78 activation, the ATF6 branch of the UPR travels to the Golgi, where it is processed by S1P and S2P proteases. Entering the activated cytosolic domain of ATF6 into the nucleus induces the expression of genes that augment the degradation and translation attenuation of misfolded proteins^64^. Hence, ATF6 appears to provide cell protection against ER stress. Nevertheless, some reports suggest that ATF6 may also have a pro-apoptotic function^65^. Moreover, CHOP and XBP1 are transactivated by ATF6^66^. Our results suggest that ASX is associated with a decrease in UTR target genes by decreasing the expression of AFT6, though reduced AFT6 expression was statistically insignificant in our work. Under severe or chronic ER stress, apoptosis is started via CHOP, as well as other mechanisms^16^. A CHOP activity was found in the ovary physiological circumstances and PCOS^66^. In the current work, ASX reduced the CHOP mRNA and protein expression levels after the intervention. By the reduced expression levels of GRP78 and CHOP, the survival branches of UPR are induced by ASX in GCs in PCOS patients. Further, it was reported that by increasing ATF4, the PERK and CHOP expression levels increased as well^67,68^. Consistent with our results, in the study of Bhuvaneswari et al., the liver tissue was protected by ASX against higher fructose and fat diet-induced damage by reducing the levels of PERK and ATF6^59^. However, Shen et al. concluded that the brain damage was attenuated by ASX in an experimental Parkinson’s disease model by reducing CHOP and GRP78 levels^69^. Wang et al. demonstrated that ethanol-induced cardiomyopathy was inhibited by ASX in mice by reducing the levels of ATF6, PERK, GRP78, ATF4, and CHOP^41^. Demir et al. demonstrated that the testicular tissue was protected by ASX against torsion/detorsion-induced injury through the antioxidant activity and suppression of endoplasmic stress. The levels of ATF6, GRP78, and CHOP markers were significantly reduced due to ASX treatment^70^. Numerous studies have shown that the activation of UPR and ER stress is implicated in chronic diseases, including inflammation, diabetes, obesity, inflammatory bowel disease, arthritis, neuromuscular inflammation disease, respiratory inflammation disease, and PCOS^16^. Investigations on type 2 diabetes and insulin resistance revealed a close relation between hyperglycemia and ER stress^61^. It was also found that androgens can increase the GRP78 expression level^62^. Higher levels of testosterone are produced in women with PCOS, along with other androgen hormones. By increasing these hormones in women with PCOS, multiple complications appear, including weight gain, acne, infertility, excessive growth of hair in the body or face, and absent or irregular menstrual periods^61^. A close relation was evidenced between signaling pathways for OS, inflammation, and ER stress^71,72^. It can be deduced that ER stress can be partially modulated by ASX antioxidant effects in PCOS patients. Although several studies have identified OS in patients with PCOS, the experimental data regarding OS markers are inconsistent^73–76^. These contradictory results could be due to small sample sizes and using different methods. It appears that antioxidant therapy might be beneficial to women with PCOS based on the OS status of these women. Our work confirmed that the FF level of TAC was increased by ASX supplementation for 60 days, while there was no considerable difference in the FF levels of MDA and SOD between the treatment and control groups. According to various animal trials, ASX can reduce OS by decreasing 8-hydroxy-2′-deoxyguanosine (8-OHdG)^77^ and MDA^78^, though it can increase antioxidant enzymes^78,79^. A limited number of clinical trials have also been performed in this regard. The results of a systematic review and meta-analysis on the antioxidant effect of ASX in humans showed that ASX might be operative in the reduction of OS as ASX may decrease the total quantity of the specific lipid peroxidation effectively (ISP and MDA) and improve plasma antioxidant capability (TAC) while increasing a definite antioxidant enzyme (SOD). However, the antioxidant effects of ASX on humans are unclear^80^. Reproductive potential is lowered in women with PCOS regardless of ovulatory state^81^ due to changes in oocytes^82^, embryo, and endometrial competence^83^, along with infertility-related co-morbidities, as well as a higher chance of pregnancy complications^84^. Virtually, every factor that is associated with PCOS also affects reproductive potential independent of PCOS. Meiotic abnormalities and poor oocyte quality are more common in women with PCOS due to the elevated OS that drives the excessive generation of ROS^81^.

Ultimately, this study discovered that administering ASX to PCOS patients before assisted reproductive technology (ART) cycles enhanced the proportion of MII and high-quality oocytes, as well as the rate of high-quality embryos. It seems that in our study, ASX might have been associated with the high-quality rate of oocyte and embryo by decreasing OS. Compared to the control group, the fertilization rate of the ASX group was not statistically greater (*P* = 0.17). In the present study, ASX had no significant effect on the fertility rate compared to the placebo. There was an increase in chemical pregnancy rates from 34.61% in the placebo group to 44.44% in the ASX group. There was also a rise in the rate of clinical pregnancy from 30.76% in the placebo group to 37.03% in the ASX group. Although the ASX group had a higher rate of chemical and clinical pregnancies, these changes were not statistically significant. The small sample size, ASX dosage, exposure length, and other factors may have contributed to the lack of statistical significance in our investigation.

One strength of our study is the coverage of all PCOS phenotypes, providing a considerably larger potential to derive generalizable results. Moreover, managing the nutritional intake and physical activity are beneficial aspects of our research. Nonetheless, this study had some limitations as well. Given that our sample size was relatively small, it may be difficult to detect small changes in response to ASX treatment. In addition, our study’s follow-up period was short; non-significant improvements in ART outcomes may be meaningful with longer follow-ups. The study was also limited by the lack of objective measurement of patient compliance; more precisely, the serum or plasma levels of ASX could not be measured in this study. However, these results may shed fresh light on the potential involvement of ASX in modulating several conditions in PCOS patients. Future studies are needed with larger sample sizes and various doses and durations to corroborate our findings. It is recommended that ASX concentrations in serum or plasma be measured as well.

It is concluded that the molecular pathways of ER stress can be modified by ASX as a natural supplement through antioxidant activity. Therefore, it can influence the expression of genes and proteins involved in the UPR in the GCs. Furthermore, OS markers may be modulated by ASX in the FF of patients. In addition, ASX may improve some of the ART outcomes for PCOS patients. Thus, ASX administration may be beneficial for PCOS patients. Eventually, the results of our study indicated that ER stress could be used as a potential therapeutic target in PCOS.

## Supplementary Information

Supplementary Information 1.

Supplementary Information 2.

Supplementary Information 3.

## Data Availability

The datasets used and analyzed during the present study are available from the corresponding author on reasonable request.

## Abbreviations

ASX: Astaxanthin
PCOS: Polycystic ovary syndrome
ER: Endoplasmic reticulum
GCs: Granulosa cells
FF: Follicular fluid
GRP78: Glucose regulated protein78
CHOP: C/EBP homologous protein
ATF4: Activating transcription factor4
ATF6: Activating transcription factor 4
XBP1: X-box binding protein 1
GAPDH: Glyceraldehyde-3-Phosphate dehydrogenase
ROS: Reactive oxygen species
NRF2: Nuclear factor E2-related factor 2
OS: Oxidative stress
UPR: Unfolding protein response
RCT: Randomized clinical trial
ICSI: Intracytoplasmic sperm injection
IBD: Inflammatory bowel disease
8-OHdG: 8-Hydroxy-2′-deoxyguanosine
BMI: Body mass index
FSH: Follicle-stimulating hormone
LH: Luteinizing hormone
Tes: Testosterone
AMH: Anti-Müllerian hormone
PRL: Prolactin
TAC: Total antioxidant capacity
SOD: Superoxide dismutase
MDA: Malondialdehyde
PVS: Perivitelline space
ZP: Zona pellucid
ART: Assisted reproductive technology
