# Supplementary material for: Astaxanthin treatment ameliorates ER stress in polycystic ovary syndrome patients: a randomized clinical trial
Source: Sci Rep. 2023 Feb 28;13:3376. doi: 10.1038/s41598-023-28956-8 (PMC9974957; doi:10.1038/s41598-023-28956-8)

**Astaxanthin treatment ameliorates ER stress in polycystic ovary syndrome patients: a randomized clinical trial**

**Masoome Jabarpour**^1^**, Ashraf Aleyasin** ^2^**, Maryam Shabani Nashtaei**^1, 2^**, Sara Lotfi**^1^**, Fardin Amidi**^1, 3^

Authors' Affiliations:

1. Department of Anatomy, School of Medicine, Tehran University of Medical Sciences, Tehran, Iran
2. Department of Infertility, Shariati Hospital, Tehran University of Medical Sciences, Tehran, Iran
3. Department of Infertility, Yas Hospital, Tehran University of Medical Sciences, Tehran, Iran

* Correspondence: Fardin Amidi, Professor of Anatomical Sciences

Address: Department of Anatomy, School of Medicine, Tehran University of Medical Sciences, Pour Sina St, Tehran, 1416753955, Iran

Telephone number: +912 31 88 556

E-mail address: [Amidifardin@yahoo.com](mailto:Amidifardin@yahoo.com)

ORCID ID: <https://orcid.org/0000-0003-1165-5232>

**Table1.** Specific primers used for real-time quantitative PCR.

| Gene | Primer |
| --- | --- |
| **GRP78** | F: CTGTCCAGGCTGGTGTGCTCT R: CTTGGTAGGCACCACTGTGTTC |
| **CHOP** | F: GGTATGAGGACCTGCAAGAGGT R: CTTGTGACCTCTGCTGGTTCTG |
| **ATF4** | F: TTCTCCAGCGACAAGGCTAAGG R: CTCCAACATCCAATCTGTCCCG |
| **ATF6** | F: CAGACAGTACCAACGCTTATGCC R: GCAGAACTCCAGGTGCTTGAAG |
| **XBP1** | F: CTGCCAGAGATCGAAAGAAGGC R: CTCCTGGTTCTCAACTACAAGGC |
| **GAPDH** | F: CGC CAG CCG AGC CAC ATC R: CGC CCA ATA CGA CCA AAT CCG |

*GRP78* Glucose regulated protein78*, CHOP* CCAAT/enhancer-binding protein homologous protein*, ATF4* activating transcription factor4, *ATF6* activating transcription factor 4, *XBP1* X-box binding protein 1*, GAPDH* glyceraldehyde-3-Phosphate dehydrogenase,

| Variables | Mean±SD Placebo (n=26) | Mean±SD Intervention  (n=27) | P-value |
| --- | --- | --- | --- |
| **Age(years)** | 30.84±4.84 | 30.36±5.16 | 0.745 |
| **BMI(kg/m2)** | 26.24±1.59 | 26.12±1.56 | 0.802 |
| **Infertility duration  (year)** | 3.386±1.93 | 4.24±2.02 | 0.147 |
| **Mean menstruation duration(day)** | 6.81±0.9 | 6.48±1.29 | 0.338 |
| **Mean menstrual cycle duration(day)** | 42.36±10.05 | 44.12±14.4 | 0.634 |
| **Baseline FSH (μIU/ml)** | 3.92± 1.11 | 4.19± 1.15 | 0.416 |
| **Baseline LH (μIU/ml)** | 9.01±3.54 | 8.85± 3.13 | 0.874 |
| **Baseline Tes (ng/ml)** | 1.18± 0.56 | 1.24± 0.55 | 0.713 |
| **Baseline AMH(ng/ml)** | 9.21±1.96 | 8.06±3.01 | 0.132 |
| **Baseline PRL (ng/ml)** | 12.36±1.8 | 13.14±2.27 | 0.203 |

**Table2.** Baseline parameters in individual group. Significance (p<0.05) was assessed by t-test.

*BMI* body mass index, *FSH* follicle-stimulating hormone*, LH* luteinizing hormone*, Tes* testosterone*, AMH* anti-Müllerian hormone*, PRL*

Prolactin

| Variables | Mean ± SD Placebo  (n = 26) | Mean ± SD Intervention  (n = 27) | P-value |
| --- | --- | --- | --- |
| **SOD(U/ml)** | 173.4 ±27.32 | 177.5 ±19.85 | 0.55 |
| **TAC (mmol  Fe2+/l)** | 0.28±0.06 | 0.32±0.04 | 0.02* |
| **MDA (μm/l)** | 2.55±0.82 | 2.18±0.89 | 0.14 |

**Table3.** Comparison of OS markers in FF of placebo and treatment groups. Significance (p<0.05) was assessed by t-test. Differences between groups; *: p<0.05. *TAC* total antioxidant capacity, *SOD* superoxide dismutase, *MDA* malondialdehyde

| Variables | Mean ± SD Placebo  (n = 26) | Mean ± SD Intervention  (n = 27) | P-value |
| --- | --- | --- | --- |
| **Number of retrieved oocytes** | 22 ± 4.88 | 21.26 ± 4.34 | 0.56 |
| **Rate of MII (mature) oocyte** | 68.81 ± 10.34 | 76.39±8.07 | 0.004** |
| **Rate of high quality oocyte** | 59.19 ± 10.45 | 67.9 ± 6.38 | 0.006** |
| **Rate of fertilization** | 77.86 ± 10.13 | 81.24 ± 7.39 | 0.17 |
| **Number of embryos** | 11.04±2.61 | 12.07±2.49 | 0.14 |
| **Rate of high quality embryo** | 63.38 ± 13.68 | 70.24 ±10.44 | 0.04* |

**Table4.** Comparison of clinical outcomes of placebo and treatment groups. Statistical significance (p<0.05) was assessed by t-test. Differences between groups; *: p<0.05, **: p<0.01


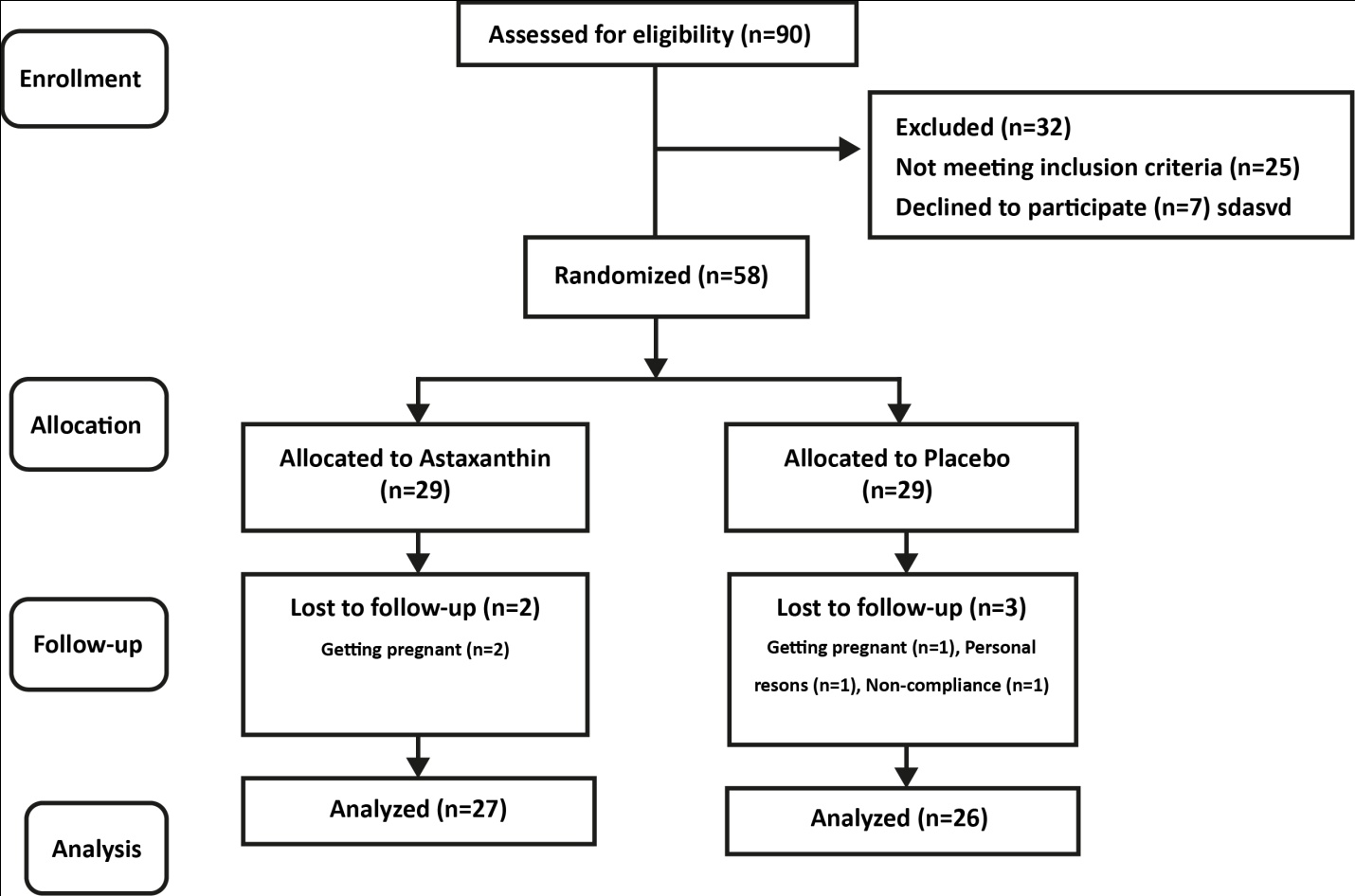


**Figure1.** Summary of patient flow through the study


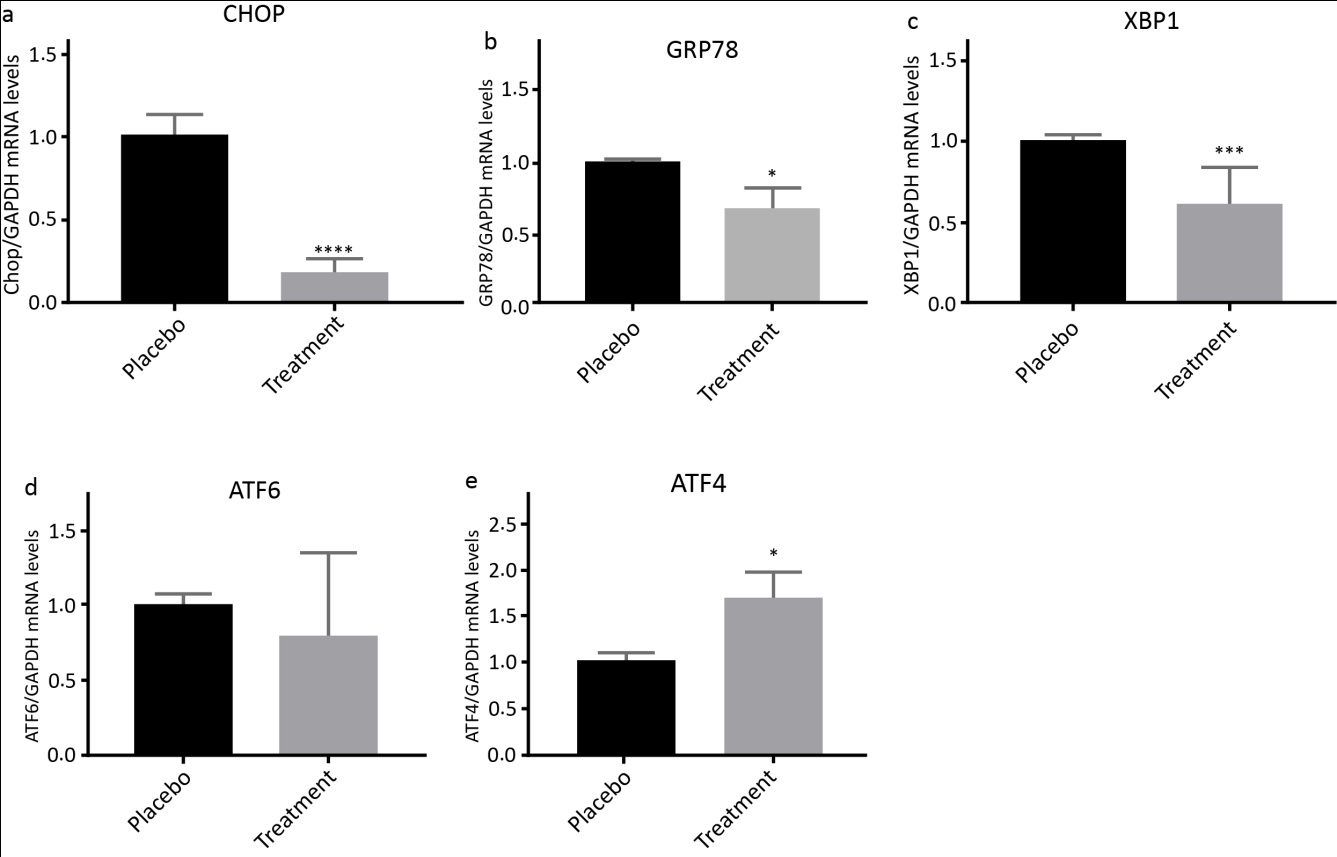


**Figure2.**The fold changes levels of CHOP (a), GRP78 (b), XBP1 (c), ATF6 (d), and ATF4 (e) in GCs of placebo and treatment groups. Statistical significance (p<0.05) was assessed by t-test. The results showed that fold changes levels of ATF4 was significantly increased in the intervention group (P < 0.05). After intervention, it was found that in the ASX group, the fold changes levels of CHOP, GRP78, and XBP1 were significantly decreased compared to the control group, while the reduction level of ATF6 was not significant between two groups (P > 0.05). P: placebo; T: treatment. Differences between groups; *: p<0.05, ***: p<0.001 and ****: p<0.0001.


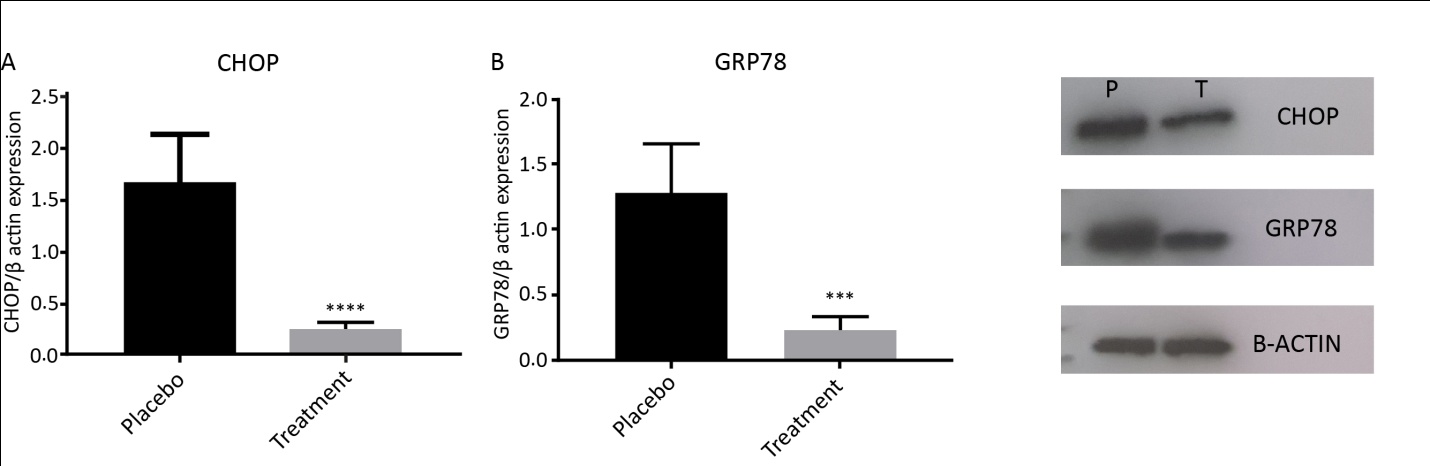


**Figure3.** The protein expression levels of CHOP and GRP78 in the GCs of placebo and treatment groups. Western blot analyzed of the protein expression of Grp78 and Chop normalized to β-actin. Following intervention, the protein expression of Grp78 and Chop significantly reduced in treatment group compared to control group. Statistical significance (p<0.05) was assessed by t-test. P: placebo; T: treatment. Differences between groups; ***: p<0.001 ****: p<0.0001.


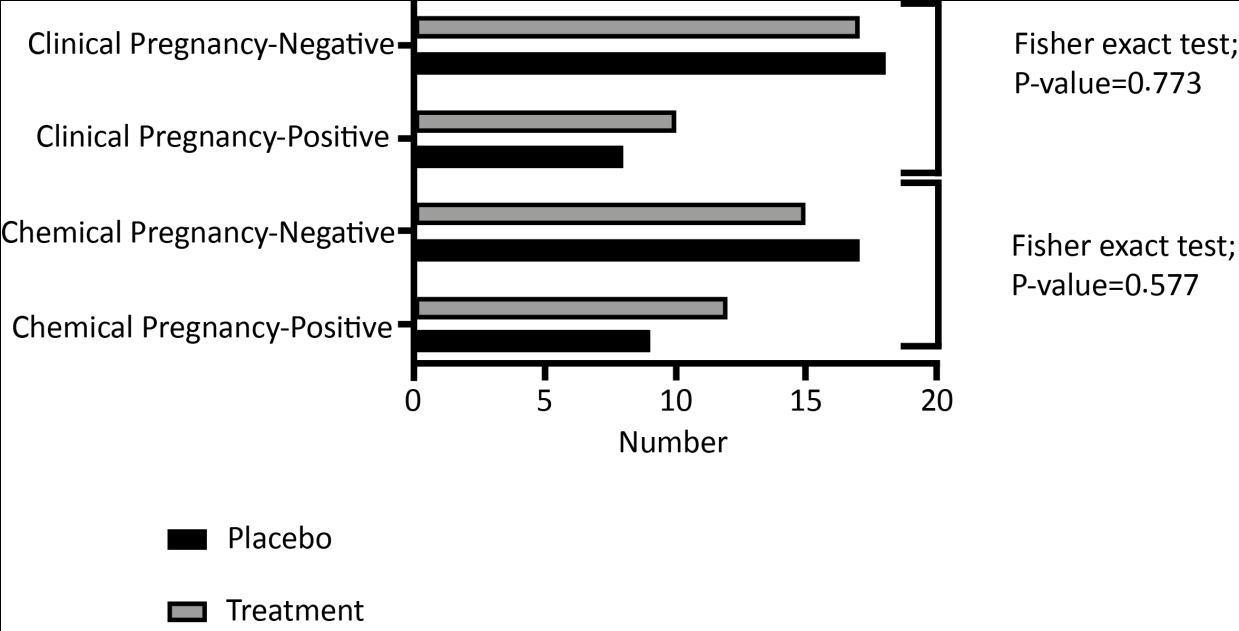


**Figure 4.**Comparison of clinical and chemical pregnancy rate between study groups. The chemical pregnancy rate was 44.44% (12/27) in the ASX group and 34.61% (9/26) in the placebo group (Fisher’s exact test; 1-sided P=0.327, 2-sided P=0.577). Moreover, the clinical pregnancy rate was 37.03% (10/27) in the ASX group and 30.76% (8/26) in the placebo group (Fisher’s exact test; 1-sided P=0.424, 2-sided P=0.773).

**Placebo=P**

**Treatment=T**


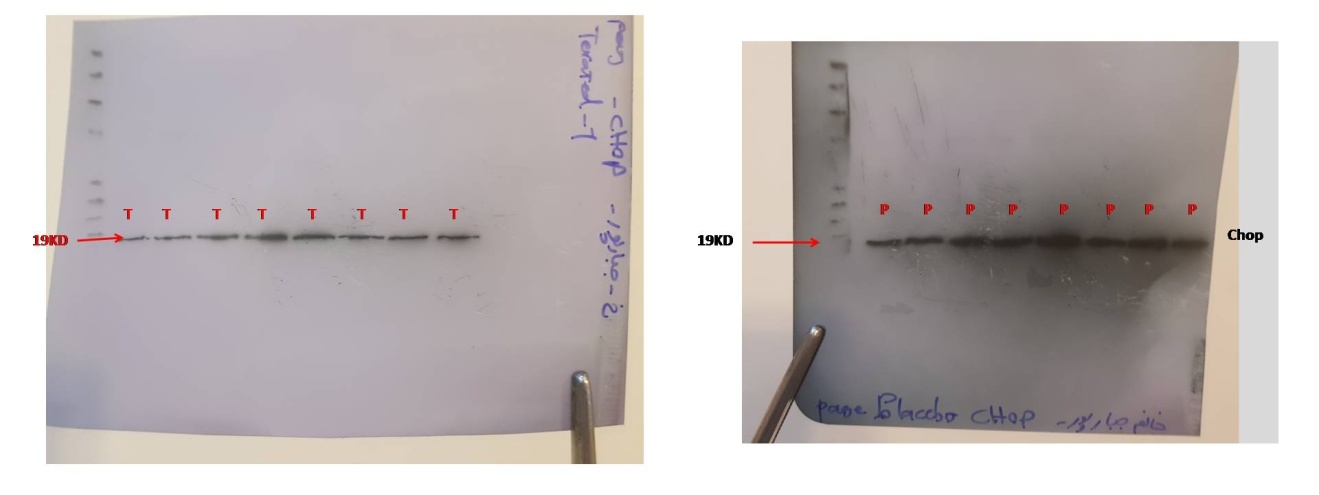

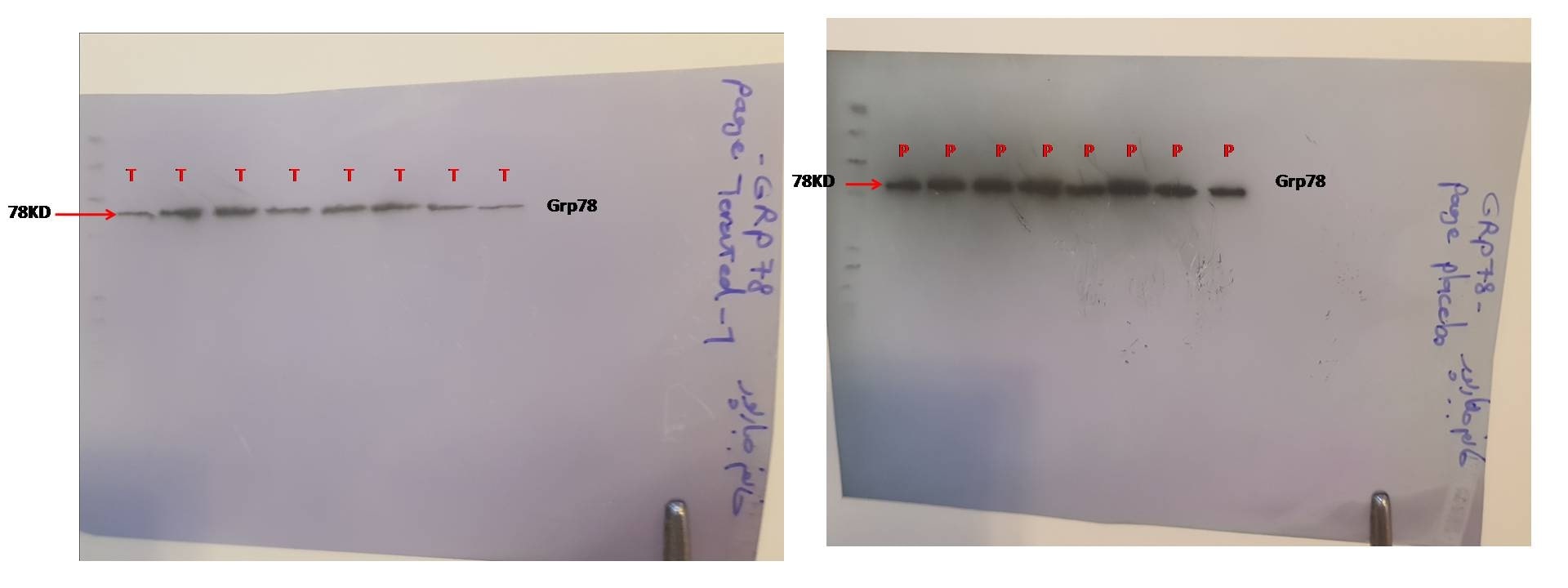


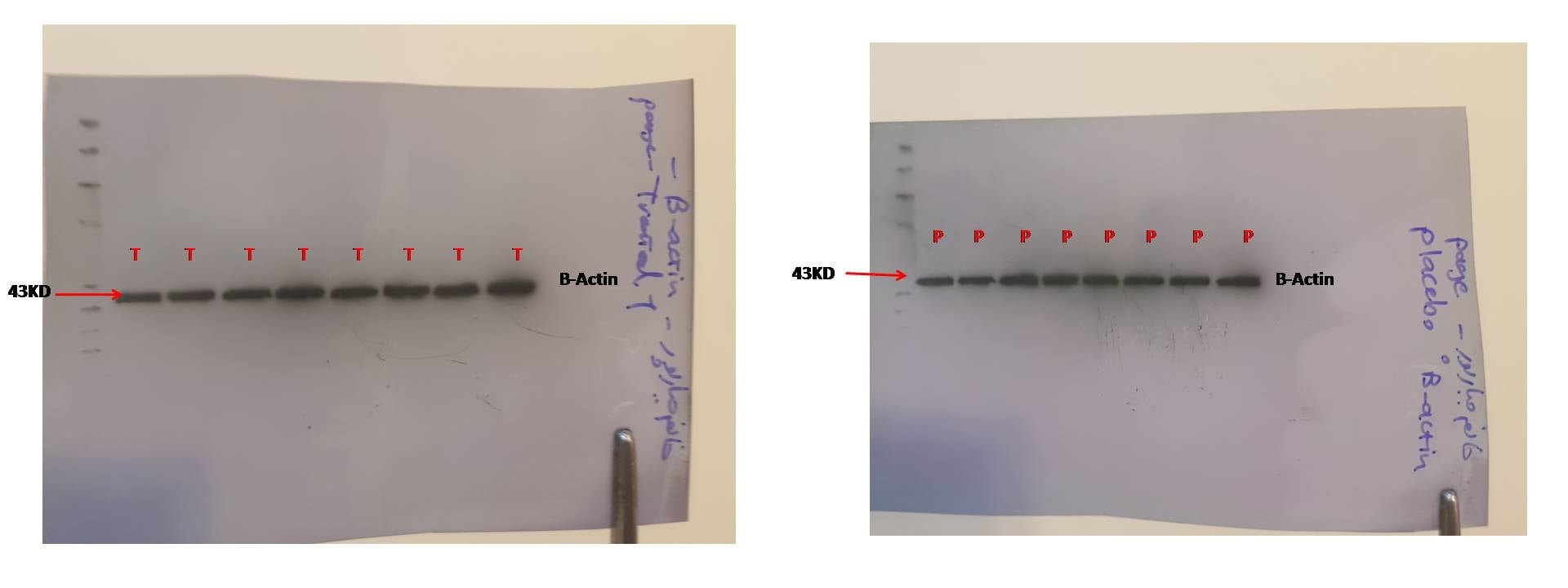


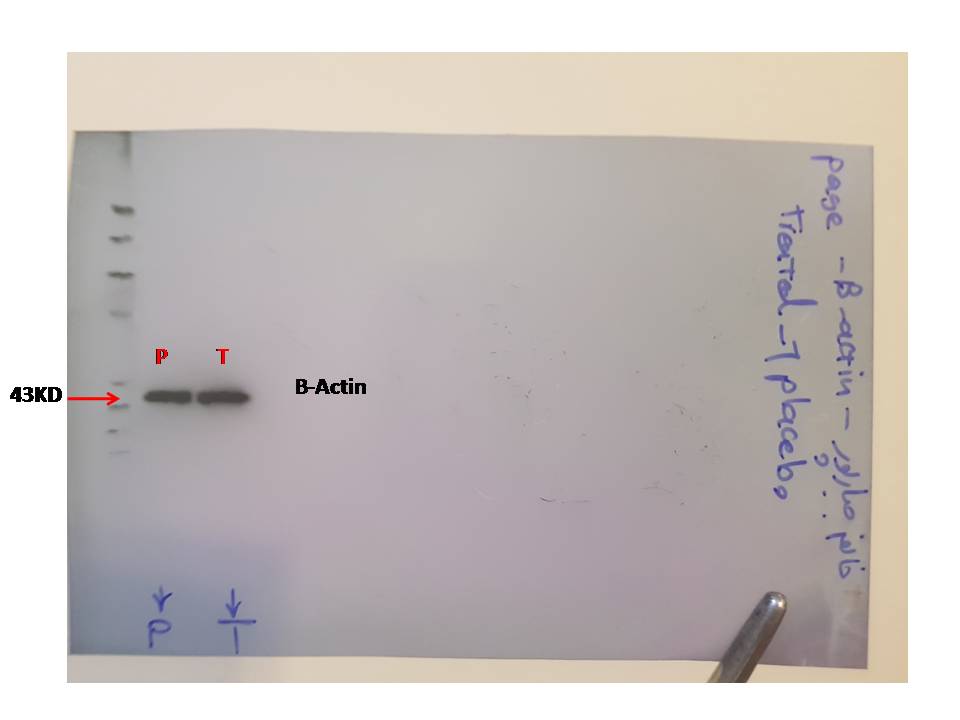

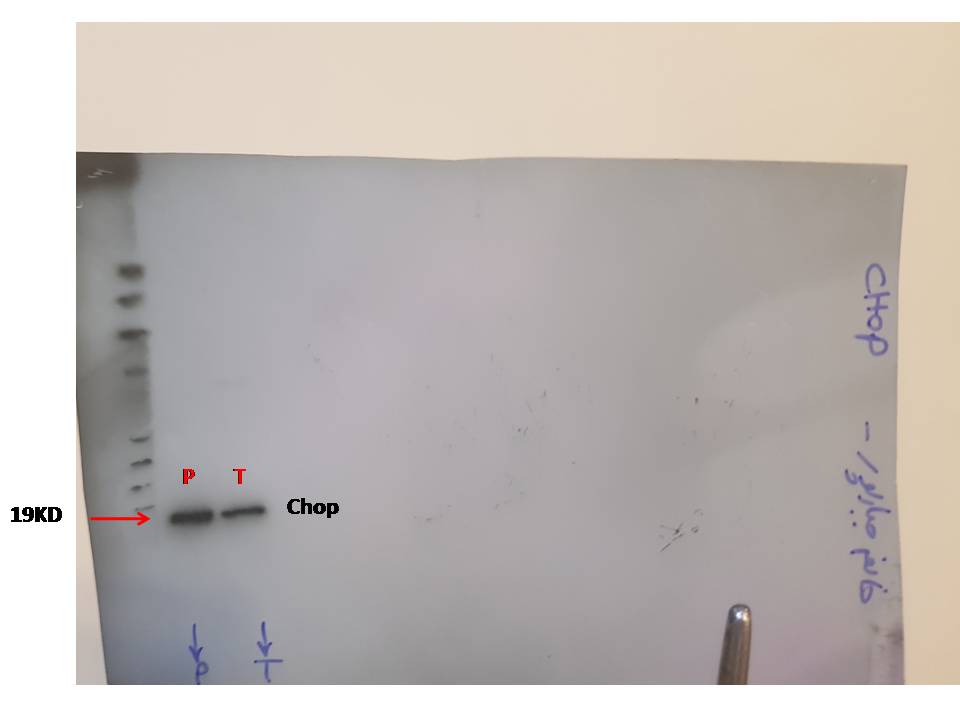

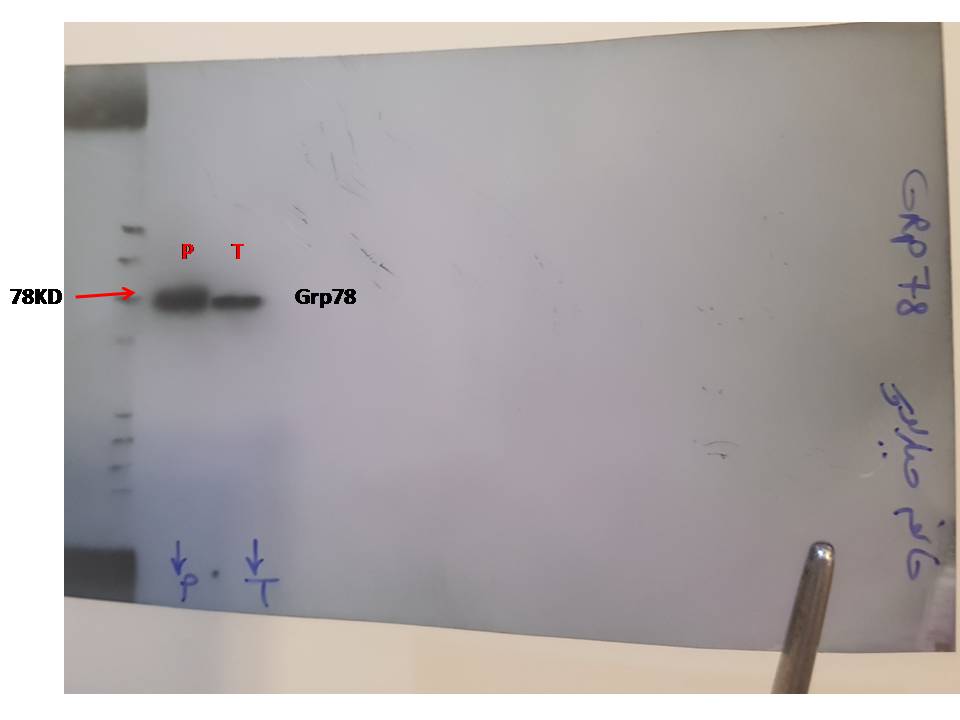

Supplement: Supplementary file 1 — Supplementary Information 1. [file 41598_2023_28956_MOESM1_ESM.docx]
